# *In vivo* and *in vitro* efficient textile wastewater remediation by *Aspergillus niger* biosorbent†

**DOI:** 10.1039/c8na00132d

**Published:** 2018-11-22

**Authors:** Shuhui Li, Jianying Huang, Jiajun Mao, Liyuan Zhang, Chenglin He, Guoqiang Chen, Ivan P. Parkin, Yuekun Lai

**Affiliations:** College of Chemical Engineering, Fuzhou University Fuzhou 350116 China jyhuang@fzu.edu.cn yklai@suda.edu.cn; National Engineering Laboratory for Modern Silk, College of Textile and Clothing Engineering, Soochow University Suzhou 215123 China; Department of Chemistry, University College London London WC1H 0AJ UK; Department of Civil Engineering, The University of Hong Kong Hong Kong China

## Abstract

In this work, the treatment of textile wastewater by a facile and high-efficiency technology using eco-friendly *Aspergillus niger* as a biosorbent was investigated. We measured physical changes (weight, size) during the formation and growth of fungus pellets and the pH values that influence the adsorption performance and biosorption mechanism. Three acid anionic dyes containing Acid Orange 56, Acid Blue 40 and Methyl Blue were chosen as model dyes to investigate batch adsorption efficiency. Two adsorption models (*in vivo* and *in vitro*) were adopted to decolorize the acid dyes. The results show that fungus pellets have excellent decoloration abilities with a high adsorption efficiency of 98% for 200 mg L^−1^ of acid dye. The pH value of the dye solution varied with the adsorption time and the dye removal efficiency greatly depended on the pH. The bioadsorption mechanism of nano-scale hyphae was revealed to be mainly due to electrostatic interactions caused by the pH change. Furthermore, the surface morphologies of the fungus after adsorption indicated that the dyes had been adsorbed on the surface of the fungus mycelia. Moreover, prepared 3D fungus/GO aerogels demonstrated superior dye removal abilities compared with fungus aerogels.

## Abstract 2

We report a facile and high-efficiency technology using eco-friendly *Aspergillus niger* as a biosorbent to treat textile wastewater. Excellent adsorption ability with nearly 98% decoloration was achieved. The bioadsorption technology could be used to fight environmental damage caused by organic or inorganic pollutants.

## Graphical abstract

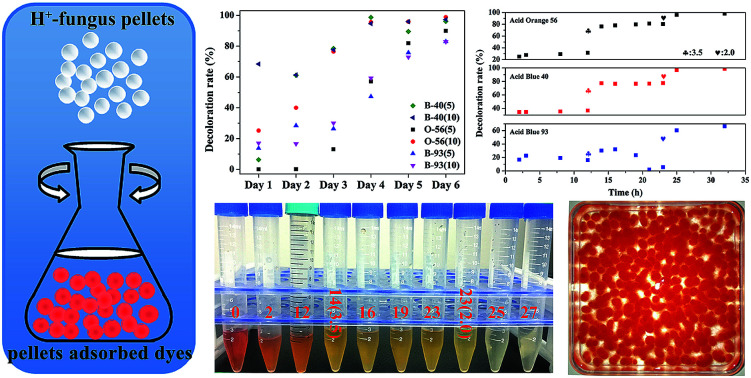

## Introduction

Dyes are organic chemical reagents that are widely used in plastics, paper, textiles, leather tanning, printing and food-making processes.^1–4^ Wastewater, especially that containing textile dyes, discharged into river systems can cause serious pollution to the environment and threaten the lives of microorganisms in the water. More than 100 000 types of commercial dyes are produced annually and over 1000 tons of dyes are annually discharged into water systems from the textile industry alone. This causes significant environmental challenges.^5^ Many materials (such as organic and inorganic film, cloth, copper mesh, aerogels and bacterials) have been applied in wastewater treatment.^6–8^ Various methods have been developed to remove dyes from wastewater and adsorption has become a commonly adopted technology.^9–12^ Acidic anionic dyes are currently used for dying natural fibers, such as wool, flax and silk, and chemical fibers, such as nylon. Most acid anionic dyes are azo-type dyes and anthraquinone-type dyes, which can pollute aquatic environments and threaten people's health and the lives of microorganisms. Therefore, textile wastewater should be strictly controlled at a permitted concentration. Before dye-containing wastewater is released into the environment, absorbents are typically applied to absorb harmful organic compounds and reduce the concentration of dyes in the wastewater.

*Aspergillus niger* is one of the most widely used fungal microorganisms due to its low cost, fast growth, easy cultivation and eco-friendly properties. In the past decades, fungi have received increasing attention as they are a rich source of degrading enzymes and possess the ability to withstand extremely harsh conditions such as low pH, fluctuating pollution load and low nutrient concentrations.^13–16^ The unique capacity of fungi to produce specific and non-specific enzymes make them very attractive for the degradation of complex organic pollutants: synthetic dyestuffs, phenols and pharmaceutical compounds.^17^ Thus, they are commonly employed in biotechnological approaches to wastewater treatment.^18–20^ There are three forms of fungal morphologies that can be found: suspended mycelia (freely dispersed filaments, nano-sized), clumps (aggregated but still dispersed) and pellets (denser, spherical aggregations).^21,22^ Fungal pellets are spherical, ellipsoidal or oval masses consisting of intertwined hyphae with sizes ranging from several hundred micrometers to several millimetres.^23–25^ Fungal pellets can offer a series of operational advantages compared to dispersed mycelia because of the ease of separating fungal biomass from complex dye solutions. Interestingly, fungal biomass can absorb dyes from wastewater regardless of whether the fungus is dry or wet. Moreover, there is no effect during the growth process of pellets if the media is mixed with organic dyes under extreme conditions. Dye decoloration with microorganisms is a cost effective and environmentally friendly method, which can be used for the removal and degradation of dye-containing effluents.

The aim of this work was to characterize the mechanism of fungal spore biosorption for the sake of obtaining a deeper insight into the potential of fungal spores as a biosorbent. In our work, we selected and cultivated a batch of *Aspergillus niger* fungus pellets as biomass adsorbent materials and evaluated the growth activity. Furthermore, we also investigated the biosorption capacity of the pellets for three typical acidic anionic dyes (Acid Blue 40, Acid Orange 56, Acid Blue 93) under various adsorption models (*in vivo* adsorption and *in vitro* adsorption). The *in vivo* adsorption method was achieved by adding dyes into the culture medium with the fungal spores and allowing the growth of fungal pellets and dye decoloration to occur simultaneously. The *in vitro* strategy involved the cultivation of fungus pellets and a biosorption process that were performed separately. Freeze-dried biosorbents were dispersed into a certain concentration of dyestuff for further adsorption.

## Experimental

### Materials and methods

#### Materials

*Aspergillus niger* (no. CCTCC AF 91006, supplied by Central South University), sucrose (AR, Sigma Aldrich), sodium nitrate, potassium dihydrogen phosphate, magnesium sulphate heptahydrate, potassium chlorate, ferrous sulfate, graphite (325 mesh, Qingdao Huatai lubricant sealing S&T Co.,), KMnO_4_, H_2_O_2_, sulfuric acid (98%), hydrochloric acid (HCl), acid dyes Everacid Blue A-2G (C.I. Acid Blue 40) and Everacid Orange N-G (C.I. Acid Orange 56) were obtained from Everlight Chemical Industry, Taiwan. Methylene Blue was purchased from Sinopharm Chemical Reagent Co., Ltd. All chemical reagents were of analytical grade and used as received without further purification.

### Experimental methods

#### Culture medium

The Czapek–Dox medium contained 30 g sucrose, 3 g sodium nitrate, 1 g potassium dihydrogen phosphate, 0.5 g magnesium sulphate heptahydrate, 0.5 g potassium chlorate and 0.01 g ferrous sulphate in 1 L of water. Before culturing *Aspergillus niger* fungus hyphae, the Czapek–Dox medium was sterilized at 121 °C under high pressure for 20 min.

#### Preparation of fungus hyphae

Several activated *Aspergillus niger* fungus hyphae were incubated in 100 mL culture medium at 37 °C with a shaking rate of 150 rpm for about 3 days. Under these conditions, the fungus mycelia could be formed in large pellet shapes. In order to obtain a fungus mycelium dispersion, vigorous agitation using a magnetic stirrer (1500 rounds per minute) was necessary. The prepared dispersed fungus hyphae were stored in a refrigerator at 4 °C for further use.

#### Preparation of fungus pellets

5 mL of dispersed fungus hyphae were added into 100 mL of sterilized culture medium. The solution was incubated at 30 °C for 72 h with a shaking rate of 150 rpm. After incubation, a large amount of fungus pellets (the diameter of single pellet is larger than 5 mm) was produced. Subsequently, the prepared fungus pellets were captured by a colander (pore size is less than 5 mm) and washed with deionized water three times so as to remove the culture medium. The collected fungus pellets were frozen and freeze-dried in a lyophilizer (−56 °C, 10 Pa) for 48 h. In order to prepare a 3D fungal aerogel, the collected fungus pellets were dispersed in water again. Before usage, the fungus pellets were treated with a blender for 3 minutes to prepare a fungal hyphae suspension. The fungus hyphae suspension was transferred into the desired container and lyophilized for 48 h to obtain a cellulose-based fungus aerogel. The mass concentration of the fungal suspension was 5 mg mL^−1^.

#### Preparation of graphene oxide (GO)

GO was obtained by using a modified Hummers method. Briefly, 1 g of graphite was added into 23 mL sulfuric acid (98%) in a 500 mL glass bottle with rigorous stirring. The glass bottle was pre-treated in an ice bath with a temperature below 5 °C. 3 g KMnO_4_ was added into the glass bottle gently and the temperature was controlled to below 20 °C. The glass bottle was then transferred into an oil bath and the reaction solution was heated to 34 °C with stirring for 2 h. After that, 50 mL water was carefully added successively to 90 °C and maintained for 10 min. Finally, another 120 mL of water and 5 mL 30% H_2_O_2_ were carefully placed into the glass bottle. The mixture quickly turned light yellow. After the reaction, the solution was vacuum filtered to collect the graphite oxide, which was rinsed with 250 mL of HCl (v/v = 1 : 10) several times to remove the metal ions. The graphite oxide solution was further purified *via* dialysis in water for 7 days. The obtained purified graphite oxide was treated with ultrasonication for 6 h to prepare the GO solution. For further usage, the GO solution was freeze dried in a vacuum dryer and prepared at the desired concentration.

#### Fungus/GO free-standing aerogel

0.2 g of dried GO pieces was dispersed into a 100 mL fungus suspension solution with a concentration of 5 mg mL^−1^. The mixture was stirred under vigorous magnetic stirring for several hours and kept at room temperature overnight. Subsequently, the mixture was poured into a 24-well cell culture plate and frozen at −80 °C for 2 h and then transferred into a vacuum drier for 3 days. Finally, the fungus/GO free-standing aerogel was prepared.

#### Batch adsorption experiment

The batch adsorption experiments were conducted by adding the adsorbents (wet fungus pellets, dried fungus pellets, fungus aerogels) into a certain concentration of acid dye solution in a conical flask. In our experiments, we poured 50 mL of 200 mg L^−1^ acid dyes into individual flasks and added 0.5 g of dry-weight fungus adsorbents. Afterward, the flask was kept in a constant temperature oscillator with a shaking rate of 150 rpm at 30 °C. The solution after adsorption was removed to measure the UV-vis spectra after a certain time period. The adsorption percentage (decoloration rate, %) and adsorption capacity (*Q*_e_, mg g^−1^) can be expressed as in eqn (1) and (2), respectively:

1 decolouration rate (%) = (*C*_o_ − *C*_e_)/*C*_o_ × 100%

2 *Q*_e_ = (*C*_o_ − *C*_e_) × *V*/*m*

where *C*_o_ and *C*_e_ (mg L^−1^) are the initial and equilibrium concentrations after adsorption and *Q*_e_ (mg g^−1^) is the equilibrium adsorption capacity. *V* (L) is the volume of acid dye solution and *m* (g) is the mass of the adsorbents.

### *In vivo* and *in vitro* adsorption of fungus hyphae

The *in vivo* culture process of fungus hyphae in acid dye solution was the same as that used for the cultivation of fungus pellets, except that 5 mg or 10 mg of acid dyes were added into 100 mL of Czapek–Dox culture medium to form the dye-containing medium. Three acid anionic dyes were applied in the adsorption experiment: Acid Blue 40 (an azo-type dye), Acid Orange 56 (a typical anthraquinone dye) and Methylene Blue (a common aromatic dye). For convenience, we defined each dye culture medium like this, for example, B-40(5) represents 5 mg Acid Blue 40 dispersed into 100 mL of Czapek–Dox culture medium. However, *in vitro* culture was conducted by adding a certain amount of dried fungus aerogel into dye solution. For the preparation of H^+^-f and HCO_3_^−^-f adsorbents, fungus pellets were soaked in 0.1 M HCl or NaHCO_3_ for 2 h, washed with water several times to remove extra reagents and finally dried in an oven. Then 0.5 g of dried adsorbent was immersed into flasks containing 50 mL 200 mg L^−1^ of each of the three different dye solutions. Afterward, these flasks were put into a constant temperature oscillator with a shaking rate of 150 rpm at 30 °C. After adsorption for a certain time, about 3 mL of the dye solution was removed to measure the UV-vis spectrum.

### Characterization

The surface morphologies of the as-prepared aerogels were conducted using a Hitachi S-4800 scanning emission microscope (SEM) at an acceleration voltage of 3 kV. For this, all samples were freeze dried and coated with a gold coating. The samples were blended with KBr and pressed into a disk, which were then examined in range of 400–4000 cm^−1^ using a Nicolet 5700 Fourier transform infrared (FTIR) spectrometer equipped with a single reflection ATR system. The resolution ratio was 4 cm^−1^. Raman spectra were obtained using a spectrophotometer (LabRAM XploRA) in the range of 4000–500 cm^−1^. The excitation wavelength was 513 nm and the acquisition time was 10 s. The chemical composition was examined using a Kratos Axis Ultra HAS X-ray photoelectron spectroscope (XPS) with an AlKα X-ray source. The excitation source was driven by 100 W power and the pressure applied in the analysis chamber was 4.0 × 10^−9^ Pa. The static water contact angles were obtained on an optical contact angle measurement Krüss DSA 100, using 6 μL deionized water droplets as the measuring liquid. UV-vis spectra were recorded on a UV-vis-NIR spectrophotometer (Cary 5000, Agilent) at room temperature with a wavelength range from 200 to 800 nm. The concentration of the dye solution was determined according to the maximum absorbance value (*λ*_max_). The pH values of the dye solutions during the whole adsorption process were examined using a Mettler-Toledo portable pH meter.

## Results and discussion

### The growth process of fungus pellets

**Scheme 1:**
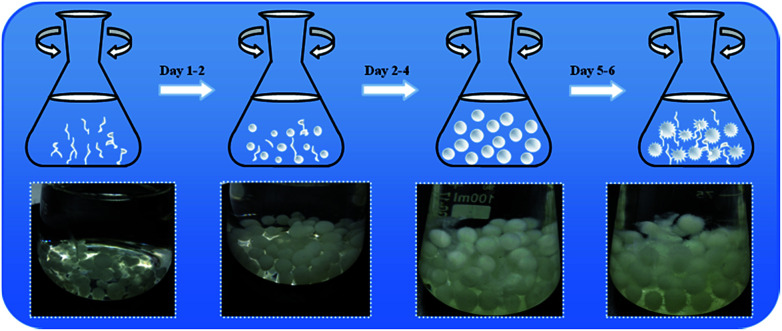
Illustration of the growth process of *Aspergillus niger* fungus pellets (upper) and the optical images in different culture periods: Day 1, Day 2, Day 4, Day 5 (bottom).

Generally speaking, three main phases have been identified for the formation and growth of filamentous fungi to pellet fungi during the whole culture process: micro-morphological growth, macro-morphological growth and fungus cell autolysis. Fungus pellets can be formed from either a single spore (non-coagulative), aggregates of spores (coagulative) or agglomerated hyphae.^26^ In our work, we selected *Aspergillus niger* as a target and researched the whole growth cycle for about one week. As shown in Scheme 1, the transformation into pellets started with the swelling and germinating phase of the spores, followed by the growth of hyphae and branching in sterilized culture medium. Once the hyphae began to branch, they intertwined with each other and gradually formed a fungal pellet. By increasing the culture time, the culture medium became exhausted and pellets began to suffer from erosion and finally broke up into filamentous hyphae due to limitations in mass transfer and oxygen. The freshly formed filamentous hyphae pieces were then used in the next growth cycle of the fungus pellets. We also obtained optical images of fungus in different culture cycles in order to better understand the growth of *Aspergillus niger*.

In addition, the influences of the fungus pellet culture time on the fungal size and mass were assessed in this experiment. As shown in Fig. S1,† after one day of cultivation, small pellets and innumerable branching hyphae emerged but the mass was relatively low. From the second day on, hyphae became intertwined with each other and gradually formed spherical fungus pellets (Scheme 1). Meanwhile, the weight and diameter of the fungus pellets exhibited a remarkable increase. With an increase in the culture time, the size and mass of the pellets grew and extended slowly. The masses of the fungus pellets were almost unchanged when cultured for 3 days compared with 4 days, which indicated that the growth of the fungus pellets reached a saturated and stable state. When the cultivation time exceeded 4 days, an autolysis phenomenon occurred and the pellet shape changed from “spherical” to “fuzzy” like a dandelion. However, the dissolved hyphae were tangled and grew new fungus spores or pellets, causing a significant increase of collected fungus biomass by weight. In addition, the pellet diameter showed almost no change when cultured between 3 and 6 days. The above results show that the formation of fungus pellets takes no more than 3 days.

### *In vivo* adsorption of fungus hyphae

**Fig. 1:**
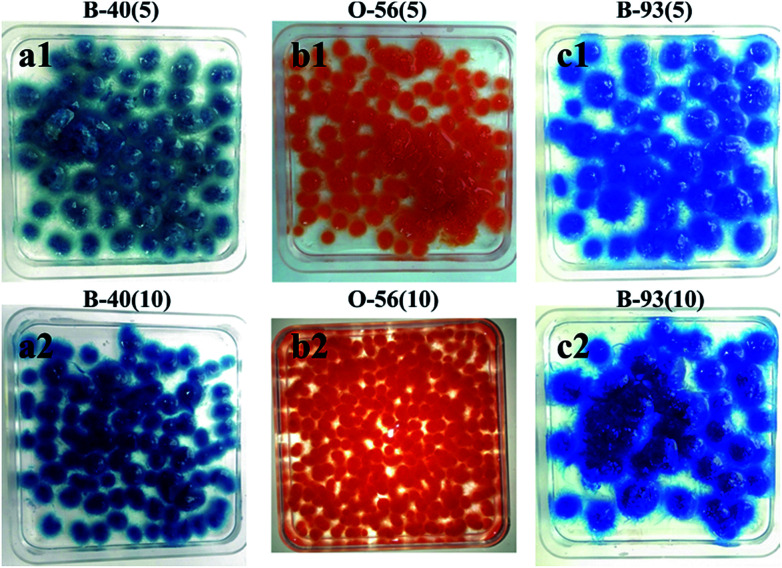
Optical images after adsorption of three different concentrations of acid dyes by *in vivo* adsorption.

The whole adsorption cycle was 6 days; fungal hyphae grew well and gradually formed fungus pellets despite the addition of dyes. At the same time, colored dyes were adsorbed throughout the formed fungus pellets (Fig. 1) and the dye culture medium became cleaner. It can be observed that the fungus pellets were filled with blue or orange dye throughout the whole biomass for various concentrations of dye, demonstrating their excellent adsorption ability.

**Fig. 2:**
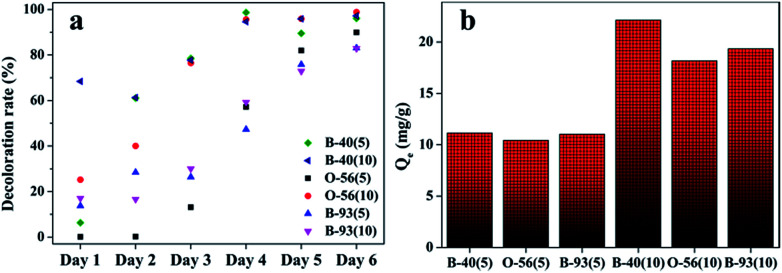
The adsorption performance of wet fungus pellets applied to acid dyes of various concentrations during the *in vivo* adsorption: decoloration rate and (a) adsorption capacity (b).

**Fig. 3:**
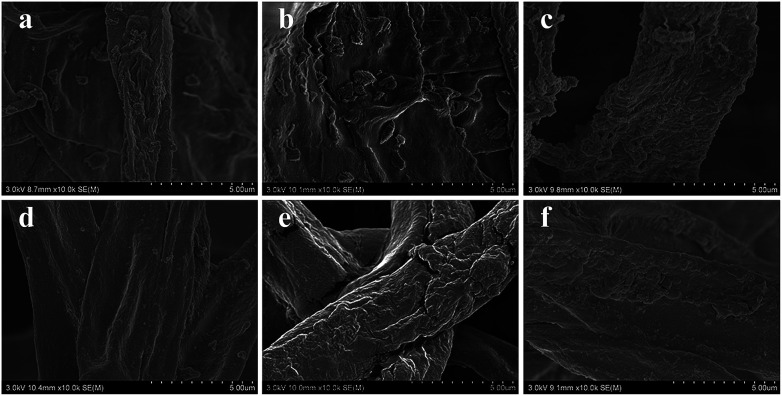
SEM images after *in vivo* adsorption of fungus hyphae with the three acid dyes under various concentrations: (a) B-40(5), (b) O-56(5), (c) B-93(5), (d) B-40(10), (e) O-56(10) and (f) B-93(10).

As shown in Fig. 2a, the decolor rates of the three acid anionic dyes increased with *in vivo* culture time. In the first three days, fungus pellets grew slowly and the yield was relatively lower, causing a slow adsorption velocity. Afterward, the yield of fungus pellets increased sharply and the adsorption ability improved significantly. Decoloration rates of above 80% were obtained for all of the various dyes after six days of cultivation. Even >98% adsorption was observed after the *in vivo* adsorption process, leaving a clean culture medium. However, low adsorption capacities for all dyes were observed, as shown in Fig. 2b, which is due to the continuous increase in the mass of fungus pellets during the *in vivo* adsorption process. The surface morphologies of the fungus pellets after adsorbing the acid dyes are shown in Fig. 3. Particles with sizes of 0.1–1 μm are coated on the surface of the fungus filaments while the surface of the fungus pellets is smooth, indicating that the dyes were adsorbed onto the fungus hyphae mainly by an electronic interaction force.

The pH changes of the dye solutions during the *in vivo* adsorption process were also recorded using a portable pH meter every 24 h. With the culture time, all dye solutions showed a decrease of pH from the initial of 4.6 to a minimum of 1.8; a decline of 1.4–2.9 in pH can be observed in Fig. S2.† The change was mainly due to the depletion of inorganic ions such as Mg^2+^, PO_4_^2−^, K^+^ and Fe^2+^ and the formation of the chemical products. The pH of the culture medium may have an effect on dye adsorption. Though high adsorption efficiencies were obtained, the long culture duration and low adsorption capacity could not satisfy the demand to rapidly purify wastewater. Therefore, the *in vivo* adsorption method does not seem to be an ideal way to adsorb dyes rapidly and efficiently. It is therefore essential to explore the adsorption mechanisms and find a new strategy that can save time and raw materials.

### *In vitro* biosorption of fungus pellets

**Fig. 4:**
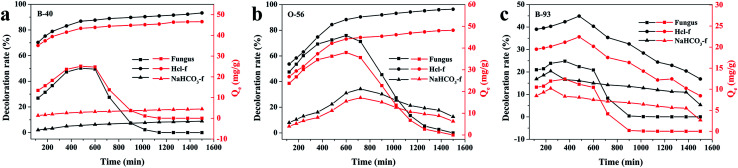
The adsorption properties of freeze-dried fungus pellets, H^+^-f pellets and HCO_3_^−^-f pellets to 200 mg L^−1^ of B-40 (a), O-56 (b) and B-93 (c).

Since the growth of fungus pellets was varied and uncontrolled during the *in vivo* biosorption, a lower adsorption capacity was obtained, as we verified before. Therefore, we investigated the adsorption performance of a quantitative controlled fungus pellet *via* a freeze-drying route. Furthermore, in order to figure out the relationship between the adsorption ability and the pH values of the dye solution, three different forms of dry-weight biomass adsorbents were applied: fungus pellets, H^+^-f and HCO_3_^−^-f. Fig. 4 shows the decoloration rates and adsorption capacities of the fungal pellets and those treated with HCl and NaHCO_3_ to the three acid dyes. Interestingly, the dried fungal pellets exhibited similar rules during adsorption of Acid Blue 40, Acid Orange 56 and Acid Blue 93 in the first 12 h and then upon desorbing. The mechanism of adsorption and the reason for this phenomenon will be discussed in detail below. However, H^+^-f pellets showed significant adsorption enhancement and achieved a high decoloration rate above 96% for Acid Blue 40 and Acid Orange 56, compared to untreated fungus pellets. Moreover, a high adsorption capacity of about 48 mg g^−1^ for these two dyes was obtained. H^+^-f pellets exhibited a low adsorption rate to Acid Blue 93, though it was better than fungus pellets. For HCO_3_^−^-f pellets, inferior adsorption performances for the three acid dyes were observed, as shown in Fig. 4. The above results indicate that acid treatment can stimulate the adsorption ability. On the contrary, an adsorption inhibition can be seen for fungal pellets treated with a base. This is due to the fact that acid treatment can provide more positive potentials and is of benefit to adsorb anionic acid dyes by a static electrical force. However, base-treated fungus pellets have more negative potentials and generate repulsive forces, preventing the as-treated fungus pellets from adsorbing acid dyes. Therefore, poor adsorption properties of HCO_3_^−^-f pellets for all acid dyes were observed.

### Effect of pH value on biosorption of dried-weight fungus pellets

**Fig. 5:**
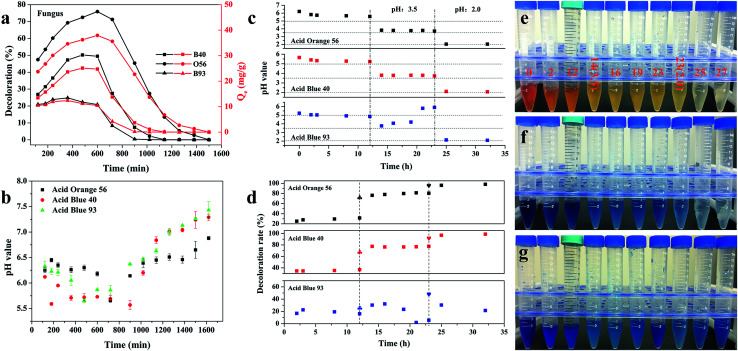
(a) The *in vitro* adsorption ability of pure fungal pellets for the three acid dyes: 200 mg L^−1^ of Acid Blue 40, Acid Orange 56 and Acid Blue 93. (b) The relationship between the pH values of the three acid dyes and adsorption time. (c) The pH changes of the three acid dyes with an increase of adsorption time. The pH was first adjusted to 3.5 after adsorption for 12 h and adjusted to 2.0 at 23 hours. (d) The adsorption rate of the three acid dyes under different pH conditions: pH unvaried (left), pH adjusted to 3.5 (middle) and pH adjusted to 2.0 (right). Two dashed lines divide the chart into three parts. The “plum blossom shape” in (d) represents the adsorption after the pH of the dye solution was adjusted to 3.5. The “heart shape” represents adsorption after the dye solution was adjusted to 2.0. (e–g) Optical images of the three acid dyes during the adsorption cycle; the numbers listed are representative of the adsorption time and the figures in brackets are the pH values of the dye solutions.

In order to investigate the reasons behind the adsorption and desorption behavior of the fungus pellets, we conducted a series of experiments. As shown in Fig. 5a, at 12 h, the fungal pellets have positive adsorption behavior; however, after this point, they exhibited negative adsorption behavior and finally recovered to almost the initial dye concentration. By monitoring the change of pH value in real time during the whole adsorption process, we found that the variation of the pH of the dye solutions was contrary to the adsorption law of fungus aerogel with adsorption duration (Fig. 5b). That is to say, pH value has a great effect on the adsorption performance. In order to verify our deduction, we adjusted the pH values of the dye solution to 3.5 and prolonged the adsorption time for another 11 h, and then changed the pH values again to 2.0 for further adsorption over 9 h. Fig. 5c shows the changes of pH values before and after adjustment for the three acid dyes. Two dashed lines divide the chart into three parts: unadjusted pH (left), pH adjusted to 3.5 (middle) and pH adjusted to 2.0 (right). Before adjusting the pH value, a slight decrease of pH was observed from 0 to 12 h (left). Meanwhile, the decoloration rate exhibited a slight increase over the first 12 h. Upon adjusting the pH value of the individual dye solution to 3.5, the pH value remained nearly steady except for when Acid Blue 93 was used, which showed a sharp increase with adsorption duration. As we predicted, the adsorption rates were significantly improved 2–4-fold for Acid Blue 40 and Acid Orange 56. For Acid Blue 93, the decoloration rate exhibited a converse trend with an increase of adsorption time compared with pH change. When the pH values were adjusted to 2.0 again, the adsorption ability reached nearly 98% for the Acid Blue 40 and Acid Orange 56.

The pH of the solution affects the ionization of the dye and the surface charge of the biomass, thus affecting their interaction.^27^ In this work, we also studied the effect of pH values on the dye solutions. As shown in Fig. S4,† the initial pH values for the Acid Orange 56, Acid Blue 40 and Acid Blue 93 were 6.80 ± 0.10, 7.03 ± 0.11 and 6.64 ± 0.08, respectively. There were no obvious changes in the color of Acid Orange 56 when the pH value was reduced using 0.1 M HCl. For Acid Blue 40, a slight decoloration was observed when the pH value was about 1.23. In contrast to Acid Blue 40 and Acid Orange 56, Acid Blue 93 is a weak acid dye. However, in the case of ammonia, zinc and other reductants, Acid Blue 93 is reduced to a colorless state. The discoloration ranges from 9.4 to 14.0 in pH (Fig. S3c†). A low concentration of Acid Blue 93 can be totally adsorbed or decomposed by fungus pellets. However, the biomass adsorbent has a lower adsorption efficiency for Methylene Blue at a high concentration, especially under acidic conditions. Though the decoloration rate increased to 48% when the pH value was adjusted to 2 for Acid Blue 93, the adsorption ability decreased gradually with the increase of adsorption time. This may indicate that the acidic conditions hamper the ability of the fungus pellets to adsorb Methyl Blue. The basic environment is likely favorable for the adsorption of Methylene Blue. Fig. 5e–g show the color changes of the three acid dyes with the increase of adsorption time as well as the decrease of pH value. Obviously, the color became fainter when the dye solution was at a lower pH; that is to say, lower pH contributed to the adsorption of the acid anionic dyes.

### Morphologies of fungus pellets after adsorption

**Fig. 6:**
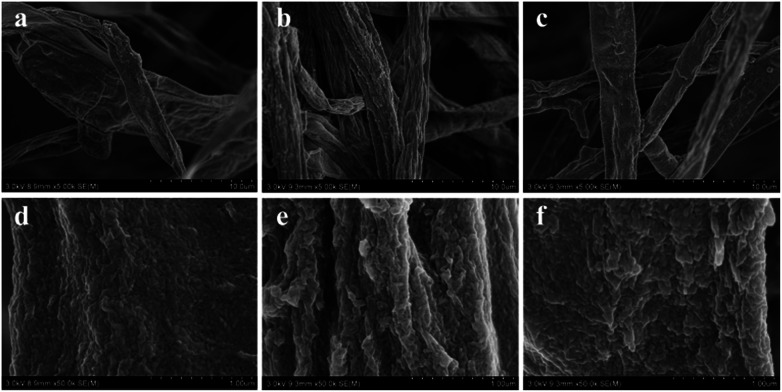
SEM images of fungus pellets after adsorption of Acid Blue 40 (a and d), Acid Orange 56 (b and e) and Acid Blue 93 (c and f).

The surface morphologies of fungal pellets after adsorbing various dyes were also characterized. Fig. 6 shows low and high-resolution SEM images of the pellets after adsorption of the three acid dyes. From these SEM images, we discovered micro/nanoscale particles were uniformly coated onto smooth fungus filaments. Meanwhile, similar surface morphologies were found on H^+^-f and HCO_3_^−^-f after biosorption. The above results reveal the high affinity of the fungus pellets to acid anionic dyes and prove them to be effective in removing dyes from wastewater.

### FT-IR analysis

**Fig. 7:**
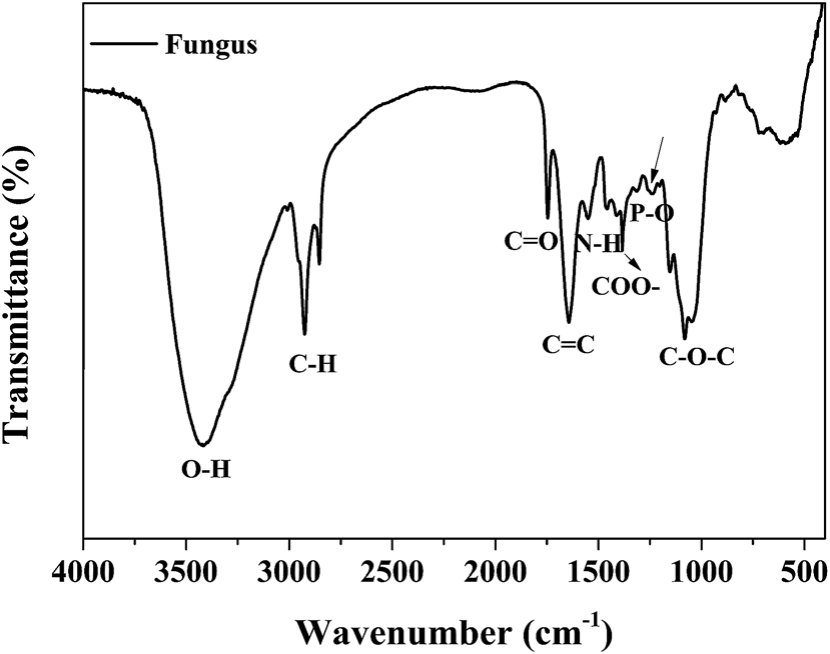
The FT-IR spectrum of fungus hyphae.

The chemical compositions of the as-cultured fungus hyphae were characterized using FT-IR measurements. As shown in Fig. 7, the peak at about 3410 cm^−1^ is attributed to hydroxyl groups. The peaks at about 2800 cm^−1^ (C–H, C–H_2_, C–H_3_) and 1720 cm^−1^ (carboxyl group) are mostly associated with the IR absorption of lipids and fatty acids. Other absorption peaks at 1620, 1540, 1440 and 1380 cm^−1^ are ascribed to the vibration of amide groups on proteins and chitin. The sharp peak at about 1100 cm^−1^ belongs to polysaccharides.^19,28^ Thus the fungus hyphae mainly consisted of polysaccharides, lipids and proteins. We also conducted EDS measurements to further confirm the chemical elements and element distribution on the hyphae (Fig. S4†).

From the EDS mapping and spectra of the fungus hyphae, we observed that the hyphae mainly consist of C, O, P and K. Au was detected from the sputtered gold layer used to improve the conductivity of the sample. The EDS mapping indicated that all of the elements were evenly distributed on the fungus hyphae. The relative C/O/P/K/Au atom ratio was found to be 64.344/34.741/0.334/0.322/0.258.

### Biosorption mechanisms

**Fig. 8:**
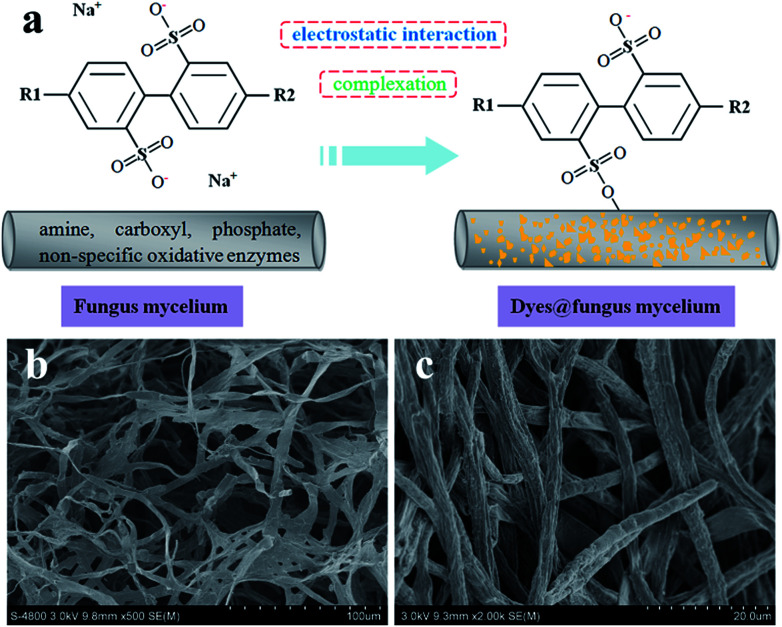
Schematic diagram of adsorption mechanism model (a), SEM images of fungus mycelia, and (b) dyes adsorbed onto fungus mycelia (c).

The biosorption mechanisms of the fungus pellets to acid anionic dyes are very complex and mainly involve biodegradation and biosorption. Complexation and electrostatic interactions are the two main mechanisms behind the biosorption. The biosorption mechanisms of the dyes are dependent upon their various chemical structures. The different functional groups of the biosorbent play different roles in the biosorption of different dyes. Below, we offer a rational interpretation of the biosorption mechanism between fungus mycelia and the acid dyes, taking Acid Orange 56 as an example (Fig. 8a). The fungal cell walls are composed of polysaccharides (chitin and chitosan), proteins, lipids and melanin with various functional groups such as amines, carboxyls and phosphate groups, which are capable of binding dye molecules.^29^ The biosorption of acid dyes involves electrostatic interactions, which could be a major factor behind dye decoloration. For some water-soluble acid dyes, they can be ionized into sodium cations and colored sulfonate anions. Therefore, the positively-charged functional groups on the fungus can attract sulfonate anions and remove them from an aqueous solution. After adjusting the pH values of the dyes with hydrochloric acid, the amino groups in the fungal biomass are protonated, and such protonated amino groups make the biosorption process of dyed sulfonate anions more easy and efficient.^30^

In addition to the electrostatic interactions, other mechanisms may be involved in the biosorption of the dyes. Fungi possess the ability to produce non-specific oxidative enzymes, which allow degradation of pollutions with highly complex structures.^17^ Fungal biosorption can be classified into two parts: (i) a passive reaction, which is non-metabolism dependent, and (ii) an active uptake, which is metabolism dependent and comprises an energy driven process.^31^ Carbon sources could supply the energy needed for active transport, contributing greatly to achieving maximum biosorption. Nutritional supplementation could maintain the production of enzymes, which is a significant factor in biodegradation.^32^ By comparing the SEM images before and after dye adsorption, we can further deduce that *Aspergillus niger* as a biosorbent has prominent biosorption properties, especially for acid anionic dyes (azo-type dyes and anthraquinone-type dyes). This biosorbent will greatly decrease the effluent concentration of wastewater from the textile industry, particularly for highly concentrated dye solutions, which is beneficial for aquatic ecosystems and human health.

### Decoloration ability of 3D fungus/GO composite aerogels

**Fig. 9:**
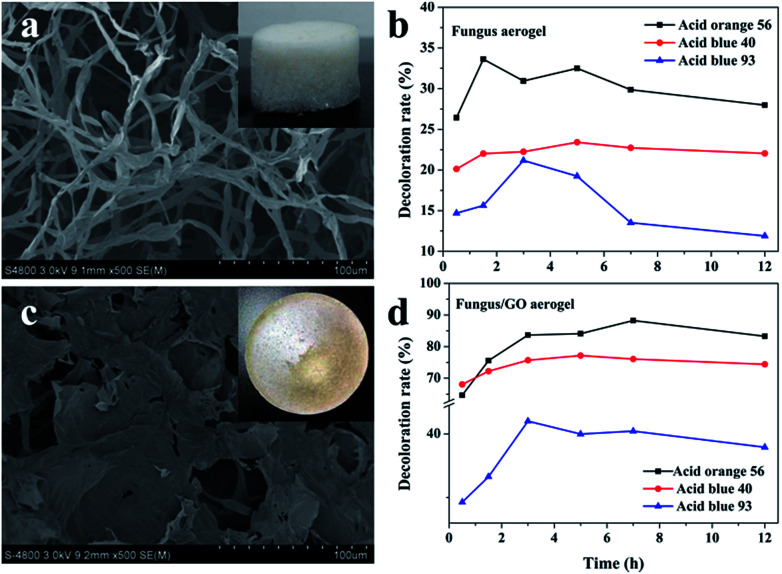
The SEM images of fungus aerogel (a) and fungus/GO aerogel (c). Insets in (a) and (c) are optical images. The decoloration ability of the fungus aerogel (b) and fungus/GO aerogel (d) to the three acid dyes.

Three-dimensional monolayer aerogel materials have attracted enormous interest in many applications due to their large specific surface area, porous structure and mechanical stability.^33–36^ Herein, we also put forward a composite fungus/GO aerogel for dye adsorption. In order to demonstrate the adsorption properties of composite fungus/GO aerogels, fungus aerogels also needed to be investigated. As shown in Fig. 9a, numerous fungus filaments with an average width of about 5 μm were observed. Such filaments intertwined with each other and accumulated layer by layer to form a 3D fungal aerogel. The weight concentration of the dried fungus filaments was 0.5%. They could not form elastic 3D self-standing aerogels below 0.5 wt% (Fig. S5†). Fungal/GO aerogels were fabricated by adding a certain concentration of graphene oxide into the fungus solution. After treatment in a freeze-dryer, the 3D self-standing fungus/GO aerogels were prepared, fungus filaments were shuttled into the graphene film and the color changed to brown (inset in Fig. 9c). It is very interesting that both the fungus aerogel and fungus/GO aerogels exhibited similar adsorption phenomena: the decoloration rate increased in the initial few hours and then decreased (Fig. 9b and d). They almost reached maximum adsorption saturation after adsorption for 3 h. The adsorption property of the fungus aerogel was similar to that of the un-modified fungus pellets, with both displaying inferior decoloration ability. However, the composite fungus/GO aerogels displayed excellent decoloration properties of around 2–3 times higher than that of the fungus aerogels.

This can be explained by the FTIR spectrum in Fig. S6.† According to the spectrum of the fungus, the peaks at about 2800 cm^−1^ (C–H, C–H_2_, C–H_3_) and 1720 cm^−1^ are mainly associated with the IR absorption of lipids and fatty acids. Some peaks located at 1620, 1540, 1440 and 1380 cm^−1^ are assigned to amide vibrations on the proteins and chitin. In addition, the sharp peaks at around 1100 cm^−1^ are caused by polysaccharides. For the FTIR spectra of GO, the absorption at peaks at about 3400, 1740, 1610, 1230 and 1000 cm^−1^ are due to hydroxyl, carbonyl, aromatic, epoxy and alkoxy groups, respectively. We can see that the intensity of oxygen-containing groups in the composite fungus/GO aerogels decreased, especially the peak at 1740 cm^−1^, indicating that the hydroxyl groups in the polysaccharides of fungus reacted with epoxy groups and formed rGO.^37,38^ During the cross-linking process between them, the functional groups changed and electron transfer occurred, which is beneficial for the biosorption of the dyes. Moreover, the GO sheet of the composite aerogel provided more surface area compared with the fungus aerogel.

### Conductivity of fungus/GO aerogels before and after carbonization

Before carbonization, fungus and fungus/GO aerogels are insulators, which cannot conduct electricity. As depicted in Fig. S7a,† an LED cannot be illuminated when conductive copper is connected to the top and bottom side of fungus/GO aerogel. However, a weak light can be observed once the copper is connected with the carbonized fungus/GO aerogel. A bright light can also be achieved by slowly compressing the aerogel, indicating that after high-temperature treatment, GO was reduced to rGO and the cellulose-based fungus became a carbon material. Therefore, carbonized fungus/GO aerogel becomes a conductor and can be used as a soft and light aerogel conductor in many applications.

## Conclusions

Fungal biotechnology for wastewater treatment has recently been of great interest in the environment decontamination of organic and inorganic pollutants. Self-assembled fungal pellets containing *Aspergillus niger* have great potential in wastewater treatment because they are easily separated from dye-containing wastewater, and exhibit good decoloration properties and strong acid/base tolerances during the culturing and adsorption processes. A batch adsorption study indicated that the fungus pellets possess efficient decoloration abilities with a high adsorption rate of 98%. An analysis of the mechanism showed that the excellent decoloration performance relies on the active groups on the fungus pellets and electrostatic interactions, including enzymes involved in cell wall degradation. The performance can be further improved by adjusting the pH values of the dye solution. Moreover, incorporating GO sheets into a fungus solution can also increase the dye adsorption. This article provides valuable insight into the management and treatment of textile effluents, especially those containing acid anionic dyes, such as azo-type dyes and anthraquinone-type dyes. We believe that micro/nano-fungus bioadsorption technology could be a potential way to fight environmental damage caused by organic or inorganic pollutions, even when the fungal biomass is inactivated.

## Conflicts of interest

The authors declare that they have no conflicts of interest.

## Supplementary Material

NA-001-C8NA00132D-s001
