# Supplementary material for: In vivo and in vitro efficient textile wastewater remediation by Aspergillus niger biosorbent
Source: Nanoscale Adv. 2018 Nov 22;1(1):168–76. doi: 10.1039/c8na00132d (PMC9473216; doi:10.1039/c8na00132d)
Supplement: NA-001-C8NA00132D-s001 [file NA-001-C8NA00132D-s001.pdf]

Electronic Supplementary Material (ESI) for Nanoscale Advances.
This journal is © The Royal Society of Chemistry 2018

Supporting information

In-vivo and in-vitro efficient textile wastewater remediation by Aspergillus niger biosorbents

Shuhui Li[a,b], Jianying Huang[a*], Jiajun Mao[b], Liyuan Zhang[d], Chenglin He[b], Guoqiang Chen[b], Ivan P. Parkin[c], Yuekun Lai[a,b*]

[a]*College of Chemical Engineering, Fuzhou University, Fuzhou 350116, China*
[b]*National Engineering Laboratory for Modern Silk, College of Textile and Clothing Engineering, Soochow University, Suzhou 215123, P. R. China*
[c]*Department of Chemistry, University College London, London, WC1H 0AJ, United Kingdom*
[d]*Department of Civil Engineering, The University of Hong Kong, Hong Kong, P. R. China*
**Corresponding Author E-mail: jyhuang@fzu.edu.cn; yklai@suda.edu.cn*

Figure Captions:

Fig. S1 The relationship between cultivation time and the growth weight and diameter of fungus spores.
Fig. S2 The interaction between pH values and cultivate time on various concentration of dyes by "in-vivo" adsorption.
Fig. S3 The optical images of three acid dyes on various pH values: (a) Orange 56, (b) Blue 40 and (c) Blue 93.
Fig. S4 The EDS analysis of fungus spore: (a-f) element mapping and (g) energy spectrum and elements proportion.
Fig. S5 The optical images of as-prepared 3D fungus aerogels with different weight concentrations of fungus hyphae: A-0.5 wt%, B-0.25 wt%, C-0.1 wt%, D-0.05 wt%. (top and bottom showed the diameter and height of as-prepared fungus aerogels, respectively)
Fig. S6 FT-IR spectrum of pure fungus aerogel, GO and composite fungus/GO aerogel.
Fig. S7 The display of conduction ability of 3D fungus/GO aerogles before (a) and after (b-d) carbonized: a LED couldn't be lighted by fungus/GO aerogel, but can be illuminated by carbonized fungus/GO aerogel.

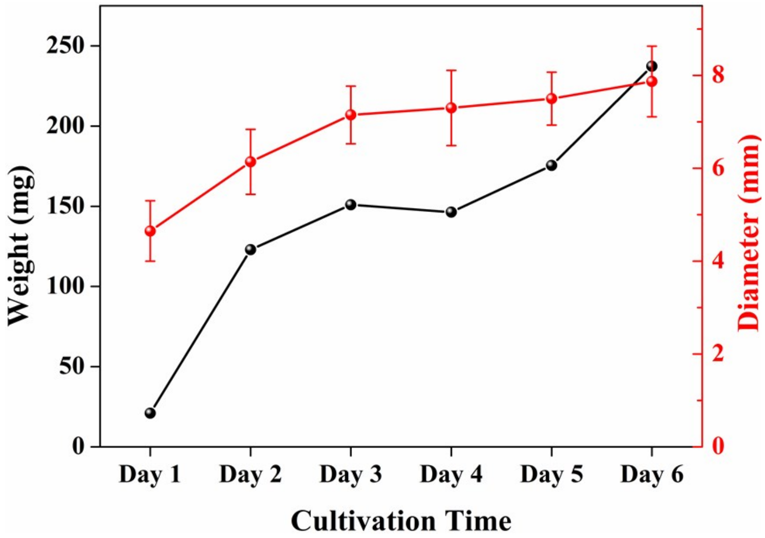

Fig. S1 The relationship between cultivation time and the growth weight and diameter of fungus spores.

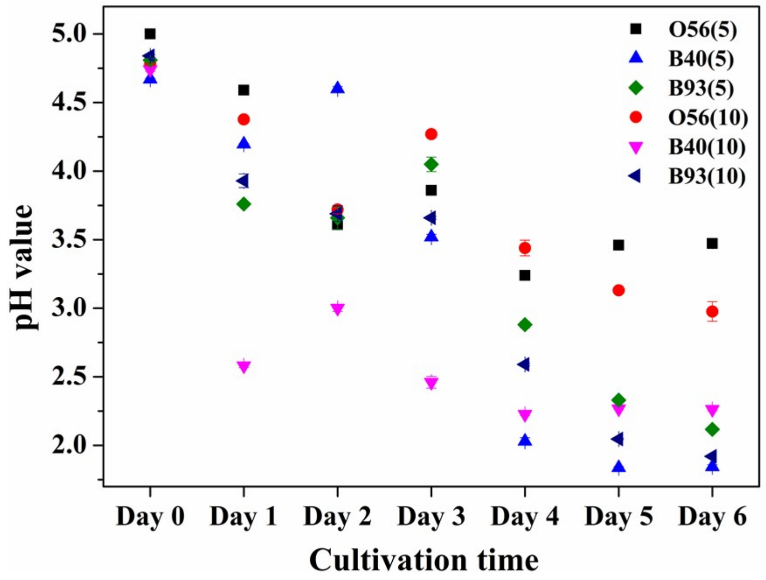

Fig. S2 The interaction between pH values and cultivate time on various concentration of dyes by “in-vivo” adsorption.

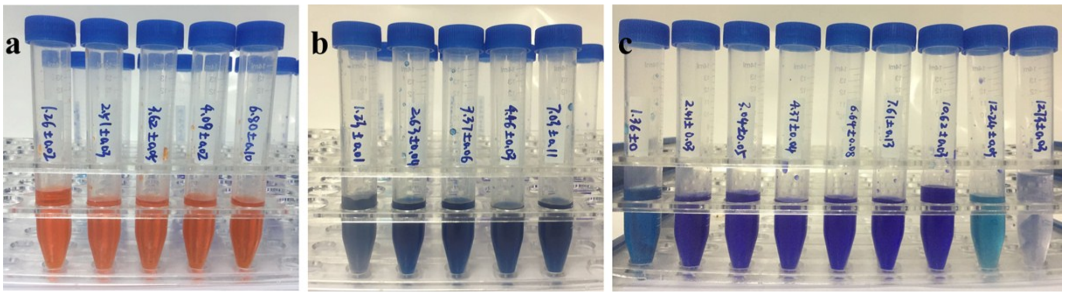

Fig. S3 The optical images of three acid dyes on various pH values: (a) Orange 56, (b) Blue 40 and (c) Blue 93.

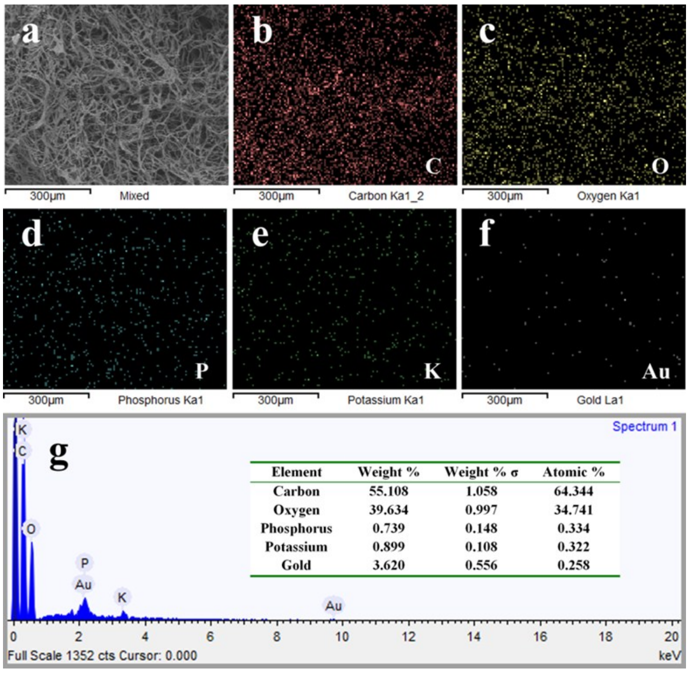

Fig. S4 The EDS analysis of fungus spore: (a-f) element mapping and (g) energy spectrum and elements proportion.

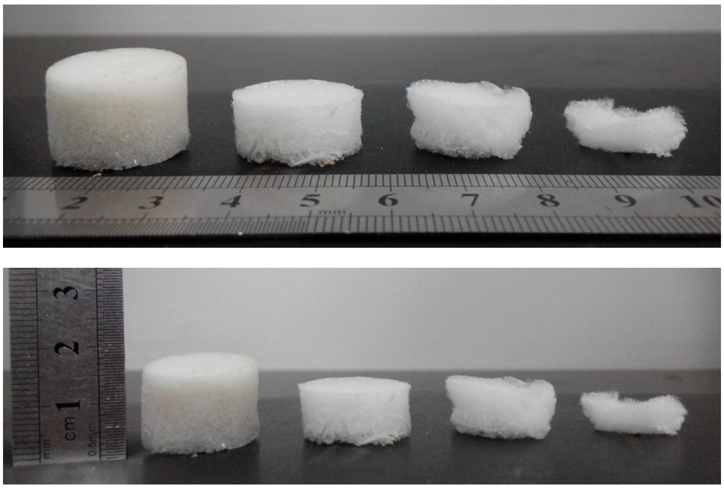

Fig. S5 The optical images of as-prepared 3D fungus aerogels with different weight concentrations of fungus hyphae: A-0.5 wt%, B-0.25 wt%, C-0.1 wt%, D-0.05 wt%. (top and bottom showed the diameter and height of as-prepared fungus aerogels, respectively)

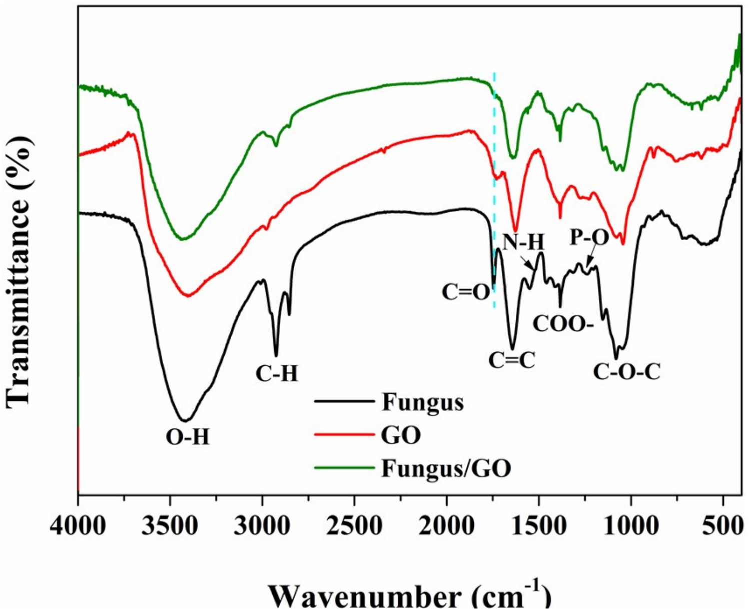

Fig. S6 FT-IR spectrum of pure fungus aerogel, GO and composite fungus/GO aerogel.

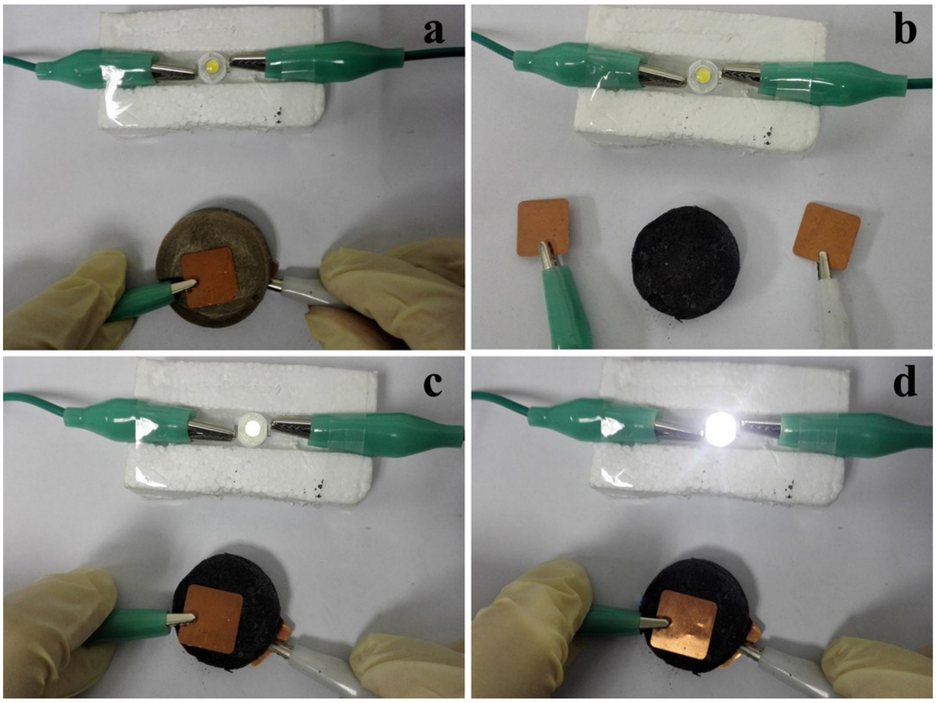

Fig. S7 The display of conduction ability of 3D fungus/GO aerogles before (a) and after (b-d) carbonized: a LED couldn't be lighted by fungus/GO aerogel, but can be illuminated by carbonized fungus/GO aerogel.
